# Missouri Veterinary Medical Association

**Published:** 1900-01

**Authors:** Horace Bradley

**Affiliations:** Secretary


### MISSOURI VETERINARY MEDICAL ASSOCIATION.

The eighth annual meeting convened December 30, 1899, in the lecture-rooms of the Kansas City Veterinary College, Kansas City, Mo. The members and veterinarians present were Drs. W. B. Welch, Charles Ellis,

L. M. Klutts, Horace Bradley, J. B. Black, S. Stewart, B. F. Kaupp R. C. Moore, Louis Medsker, J. S. Buckley, James L. Otterman, C. M. McFarland, Charles Canfield, J. S. Grove, C. A. Monney, D. W. Elliott, H. T. Doak, and H. G. Patterson.

An interesting clinic, conducted by Dr. R. C. Moore, was held in the operating-room of the college at 1 P.M., lasting five hours. It was enjoyed by all present, and it was the unanimous decision that clinics should be one of the features of our meetings in the future.

The evening session opened at 8 o'clock, with President Dr. L. M. Klutts in the chair. Dr. S. Stewart and Dr. W. B. Welch were elected members. The application of Dr. V. J. Andre, of St. Genevieve, was received too late, but will be acted upon at the next meeting.

The following officers were elected for the ensuing year: Dr. H. Bradley, Windsor, President; Dr. J. W. Connaway, Columbia, Vice-President; Dr. B. F. Kaupp, Kansas City, Mo., Secretary-Treasurer.

Dr. S. Stewart, chairman of the Committee on Legislation, made a report, which was received and the committee discharged. Dr. Charles Ellis, of St. Louis, moved that the President appoint a committee to work for the legislation bill, the committee to consist of one member from each Congressional district; seconded, and motion carried.

A good and interesting programme was rendered, and all joined in a lively discussion of the papers. Many interesting cases were reported. The report on the use of black-leg vaccine proved that it has been very efficient in the hands of veterinarians in the State.

At 12 o'clock a motion was carried to adjourn to meet in St. Louis, October, 1900.

HORACE BRADLEY, V.S.,
Secretary.

